# Proanthocyanidins Extracted from *Rhododendron pulchrum* Leaves as Source of Tyrosinase Inhibitors: Structure, Activity, and Mechanism

**DOI:** 10.1371/journal.pone.0145483

**Published:** 2015-12-29

**Authors:** Wei-Ming Chai, Rui Wang, Man-Kun Wei, Zheng-Rong Zou, Rong-Gen Deng, Wei-Sheng Liu, Yi-Yuan Peng

**Affiliations:** 1 College of Life Science and Key Laboratory of Small Functional Organic Molecule, Ministry of Education, Jiangxi Normal University, Nanchang, Jiangxi 330022, People’s Republic of China; 2 Key Laboratory of Poyang Lake Wetland and Watershed Research, Jiangxi Normal University, Nanchang, Jiangxi 330022, People’s Republic of China; University of Alabama at Birmingham, UNITED STATES

## Abstract

The objective of this study was to assess the structure, anti-tyrosinase activity, and mechanism of proanthocyanidins extracted from *Rhododendron pulchrum* leaves. Results obtained from mass spectra of matrix-assisted laser desorption/ionization time-of-flight mass spectrometry (MALDI-TOF MS) and high performance liquid chromatography electrospray ionization mass spectrometry (HPLC-ESI-MS) revealed that proanthocyanidins were complex mixtures of procyanidins, prodelphinidins, propelargonidins, and their derivatives, among which procyanidins were the main components. The anti-tyrosinase analysis results indicated that the mixtures were reversible and mixed competitive inhibitors of tyrosinase. Interactions between proanthocyanidins with substrate (L-tyrosine and 3,4-dihydroxyphenylalanine) and with copper ions were the important molecular mechanisms for explaining their efficient inhibition. This research would provide scientific evidence for the use of *R*. *pulchrum* leaf proanthocyanidins as new novel tyrosinase inhibitors.

## Introduction

Tyrosinase (monophenol, dihydroxyphenylalanine: dioxygen oxidoreductase), also known as polyphenol oxidase, is a copper-containing oxidase widely existing in plants, animals, and microorganisms [1]. It is an important enzyme that is responsible for melanin biosynthesis, browning in fruits and vegetables, and insect development in organisms [2–4]. The enzyme can catalyze the hydroxylation of monophenols (monophenolase activity) and the subsequent oxidation of o-diphenols to the corresponding o-quinones (diphenolase activity) [2]. The quinones are cyclized and polymerized to produce colored pigments [3,5]. The color change caused by browning reactions generally results in losses of nutritional quality and economic value and therefore becomes a major problem in the food industry. What’s more, over upregulated tyrosinase expression or activity can result in melanoma malignum and pigmentation disorders (e.g. age-related skin hyperpigmentation, lentigo senilis, urticaria pigmentosa) [6,7]. Hence the inhibition of tyrosinase activity (melanogenesis) appears as a rational adjuvant approach to the therapy of melanoma and pigmentation disorders [6–9]. In addition, this enzyme plays important roles in insect developmental processes, such as cuticular tanning, scleration, wound healing, production of opsonins, and nodule formation for defense against foreign pathogens [10]. Therefore, tyrosinase inhibitors are quite important in the area of medicinal, food, agriculture, and cosmetic industry. Bioactive compounds extracted from plants have attracted more and more attentions because of their efficient inhibitory activity on the tyrosinase [11,12]. In this study, *R*. *pulchrum* proanthocyanidins were therefore used as source of tyrosinase inhibitors.

**Fig 1:**
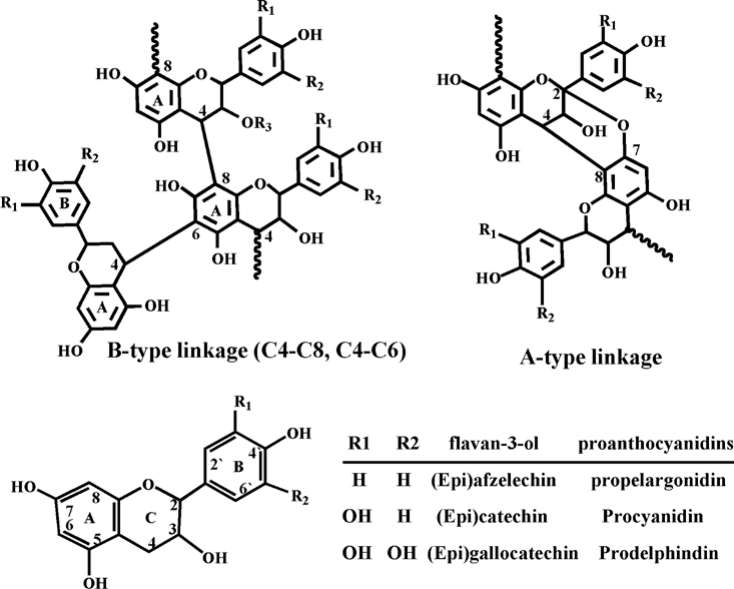
Chemical structure of proanthocyanidins and flavan-3-ol monomer units.

Proanthocyanidins are a class of bioactivity material wildly existed in plants. They are oligomers and polymers of flavan-3-ol that are linked through B-type and A-type linkages [13] (**Fig 1**). These compounds possess structural heterogeneity: monomer units, distribution of polymerization degree, interflavan linkage, and substituents [13–15]. Because of the complexity and diversity, the characterization of their structures is still very challenging. In this study, high performance liquid chromatography electrospray ionization mass spectrometry (HPLC-ESI-MS) and matrix-assisted laser desorption/ionization time-of-flight mass spectrometry (MALDI-TOF MS) analyses were employed to characterize the structures of these compounds.

*R*. *pulchrum* is an evergreen shrub, which possesses high appreciation value and medicinal efficacy. Recently, the flavonoids extracted from its leaves were characterized by HPLC-MS and were mostly the flavonoid glycosides with quercetin as the aglycone [16]. However, there were no reports on the structure and activity of proanthocyanidins. In this study, to make full use of this plant, proanthocyanidins were extracted and purified, and their structures, anti-tyrosinase activity as well as mechanism were studied to provide scientific evidence in the development of natural tyrosinase inhibitors.

## Materials and Methods

### 2.1 Plant Material and Sample Preparation

The fresh leaves of *R*. *pulchrum* were collected from the campus of Jiangxi Normal University (Nanchang, China) in June 2011 and were uniform in shape and size without physical damages or injuries. They were washed and immediately freeze-dried in the laboratory. The leaves were then ground by using a cutting mill (model BL301D5; Saikang, China) and sieved by a 50 mesh sieve to obtain fine powder. The powders were stored at −20°C before further analysis.

### 2.2 Chemicals and Materials

All analytical grade solvents (acetone, petroleum ether, ethyl acetate, and methanol) for the extraction and purification were purchased from Sinopharm (Sinopharm, Shanghai, China). HPLC grade acetonitrile, dichloromethane, and methanol for analytical HPLC-ESI-MS were also obtained from Sinopharm. L-tyrosine, 3,4-dihydroxyphenylalanine, Mushroom tyrosinase, Sephadex LH-20, HPLC standards, benzyl mercaptan, trifluoroacetic acid, Amberlite IRP-64 cation-exchange resin, cesium chloride, and 2,5-dihydroxybenzoic acid were purchased from Sigma-Aldrich (St. Louis, MO, USA).

### 2.3 Extraction and Purification of the Proanthocyanidins

Acetone/water (70:30, v/v) was used as the solvent for extraction. Petroleum ether and ethyl acetate were selected as extractant to eliminate chlorophyll, lipophilic compounds, and low molecular phenolics. The remaining fraction was then poured into a Sephadex LH-20 column (50 × 1.5cm i.d.) which was eluted with methanol-water (50:50, v/v) and then acetone-water (70:30, v/v) and the latter were reserved. At last, purified tannins were obtained after removing acetone and freeze-dried.

### 2.4 MALDI-TOF MS Analysis

The MALDI-TOF MS analysis was carried out by a Bruker Reflex III (Germany). The irradiation source was a pulsed nitrogen laser with a wavelength of 337 nm, and the duration of the laser pulse was 3 ns.

### 2.5 Reversed-phase HPLC-ESI-MS Analysis Followed by Thiolysis Reaction

Proanthocyanidins were degraded in the presence of benzyl mercaptan, and then the degradation products were injected into an Agilent 1200 system (Agilent, Palo Alto, CA, USA) interfaced to a QTRAP 3200 (Applied Biosystems, Foster, USA) with a 250 mm× 4.6mm i.d. 5.0mm Hypersil ODS column (Elite, Dalian, China). The elution system was: 0–45 min, 12–80% B (linear gradient); 45–50 min, 80–12% B (linear gradient). The column temperature was 25°C and the flow rate was 1 mL/min.

### 2.6 Normal-phase HPLC-ESI-MS Analysis

Normal-phase HPLC-ESI-MS analysis was conducted in a 250mm × 4.6mm i.d., 5 μm Silica Luna (Phenomenex, Darmstadt, Germany). The HPLC equipment consisted of an Agilent 1200 liquid chromatograph system. The eluant, linear gradient, and conditions for eluting were carried out on the basis of the procedure described in previous report [17]. In addition, instrument parameters for MS analysis were as follows: ions spray voltage, 4.5 kV; declustering potential, 50 V; entrance potential, 10 V; curtain gas, 20 psi; source temperature, 400°C.

### 2.7 Enzyme Assay

The procedure for enzymatic analysis was carried out in compliance with previous study [18]. In brief, L-tyrosine was selected as substrate for monophenolase activity assay and 3,4-dihydroxyphenylalanine was chosen as the substrate for diphenolase activity of tyrosinase. The final concentrations of tyrosinase were 33.33 and 3.33 μg/mL for the analysis of monophenolase and diphenolase reaction, respectively. Absorption (475 nm) was detected by using a Beckman UD-800 spectrophotometer (USA). The initial slope of the kinetic curve was used as an indicator of tyrosinase activity. Their results were expressed with *IC*_*50*_ value (μg/mL). An inhibitory mechanism assay was carried out by changing the enzyme concentration in the reaction medium. The plots of the remaining enzyme activity versus the concentrations of enzyme in the presence of inhibitors with diffierent concentrations gave a family of straight lines. If the inhibition was reversible, all straight lines intersect at the origin; when the inhibition was irreversible, these straight lines are parallel. The inhibition type depended on the Lineweaver-Burk plot.

### 2.8 Fluorescence Quenching Analysis

The fluorescence assay of tyrosinase and proanthocyanidins was measured by a Varian Cary Eclipse fluorescence spectrophotometer. The reaction medium (1 mL) contained 100 μL of tyrosinase solution, 800 μL of 50 mM sodium phosphate buffer (pH 6.8), and 100 μL of sample solution with different concentrations. The experiments were performed with an excitation wavelength of 285 nm at a constant temperature of 25°C.

The fluorescence assays of L-tyrosine/3,4-dihydroxyphenylalanine and proanthocyanidins were also carried out through a Varian Cary Eclipse fluorescence spectrophotometer. In brief, L-tyrosine (12 mM) or 3,4-dihydroxyphenylalanine (0.5 mM) solution (100 μL) was added into 50 mM sodium phosphate buffer (pH 6.8), and sample solution was then added (the final medium was 1 mL). The mixure were preincubated for 30 s before fluorescence spectra measurements with an excitation wavelength of 285 nm.

### 2.9 Determination of Cu^2+^ Chelating Activity

Copper sulfate solution (100 μL) with different concentrations (12.5, 25, 50 μM) and sample (100 μL, 3 mg/mL) solution were mixed with sodium phosphate buffer (50 mM, pH 6.8). The final mixture (1 mL) was kept at 25°C for 1 min prior to fluorescence quenching analysis.

### 2.10 Molecular Docking of Tyrosinase with the Ligands

In this research, molecular operation environment 2010 software (MOE) was used for protein-ligand docking. The structure of the oxy tyrosinase from *Streptomyces castaneoglobisporus* was used as the initial model for docking simulations. The 3D structures of the ligands were prepared with Chembiodraw Utra 12.0. Before docking, the structure models of the protein and ligands were energy-minimized using the energy minimization module of the MOE. At the same time, hydrogens were added to the models of the protein and ligands with the protonate 3D module. For molecular docking, the refinement module was set to forcefield and retain of first and second scorings were set to 20 and 10, respectively. The MM/GBVI binding free energy scoring was used to rank the docking poses. A more negative value reflects a stronger interaction. The docked conformation which had the highest score was selected to analyze the mode of binding.

## Results and Discussion

### 3.1 MALDI-TOF MS Analysis

**Fig 2:**
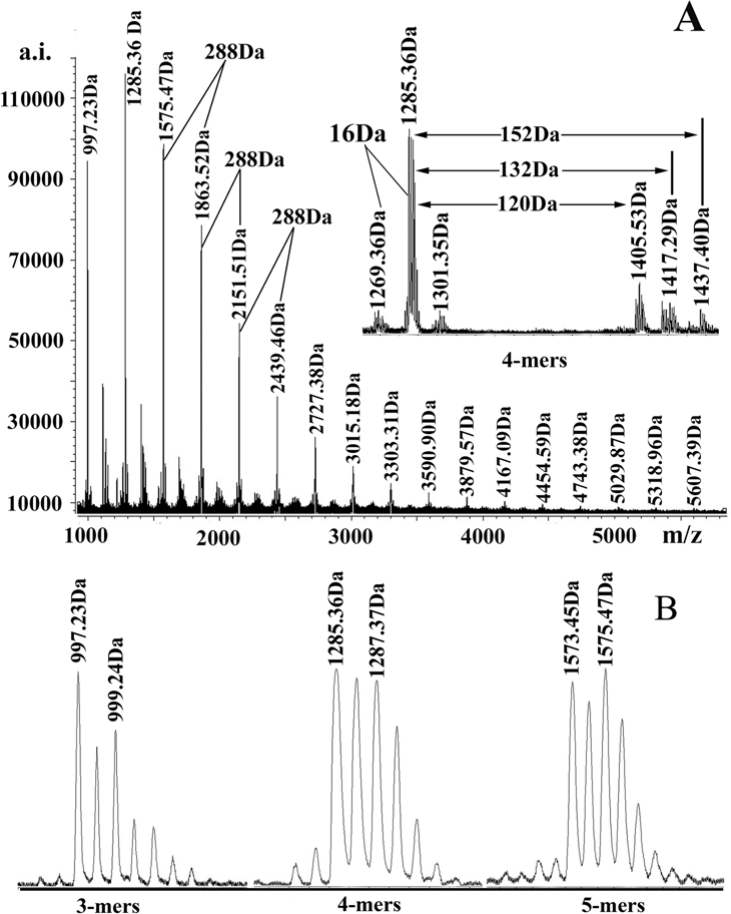
A, MALDI-TOF positive-ion (Cs^+^) reflectron mode mass spectra of the proanthocyanidins from *R*. *pulchrum* leaves. B, Magnified MALDI-TOF mass spectra of 3-mers, 4-mers, and 5-mers.

MALDI-TOF MS has proved itself a sensitive and powerful tool for the characterization of plant proanthocyanidins which exhibit structural heterogeneity [19–21]. **Fig 2A** showed the MALDI-TOF mass spectra of *R*. *pulchrum* proanthocyanidins which were recorded as Cs^+^ adducts in the positive ion reflection mode. And **S1 Table** provided their observed and calculated masses by MALDI-TOF-MS. The mass increased equably from 997.23 Da (3-mers) to 5607.39 Da (19-mers) at a distance of 288 Da, which corresponded to the addition of one epicatechin/catechin monomer unit. This revealed that the main structural unit of *R*. *pulchrum* proanthocyanidins was epicatechin/catechin. For each multiple, substructures with more than or less than 16 Da were also found in spectra when they were enlarged (**Fig 2A, S1 Table**). These masses had been identified as heteropolymers of repetitive flavan-3-ol units, which indicated the presence of epigallocatechin/gallocatechin and epiafzelechin/afzelechin. These findings demonstrated the coexistence of procyanidin, prodelphinidin, and propelargonidin in *R*. *pulchrum* proanthocyanidins. Besides the series of peaks described above, 152 Da and 132 Da mass distances following the main set of peaks were also detected (**Fig 2A**). The first series (152 Da) produced by the addition of one galloyl group at the heterocyclic C-ring [22]. And the 132 Da mass distance may be produced by the substitution of a pentoside [22] or by synchronous attachment of two Cs^+^ and absence of a proton [M +2Cs +2H]^+^ [23]. Specifically, a series of peaks (120 Da mass distances between the main set of peaks) were found in the mass spectrum. They may be new proanthocyanidins or their derivative. Additionally, the A-type interflavan linkages (2 Da less than B-type) were also detected in the main set of peaks (**Fig 2B)**, which illustrated the nature of interflavan bonds including A-type and B-type linkages. These results demonstrated structural heterogeneity of proanthocyanidins in the *R*. *pulchrum*. The structures of *R*. *pulchrum* proanthocyanidins were therefore successfully characterized by using MALDI-TOF MS method for the first time.

### 3.2 Proanthocyanidins Analysis by Reversed-phase HPLC-ESI-MS after Thiolysis Reaction

**Fig 3:**
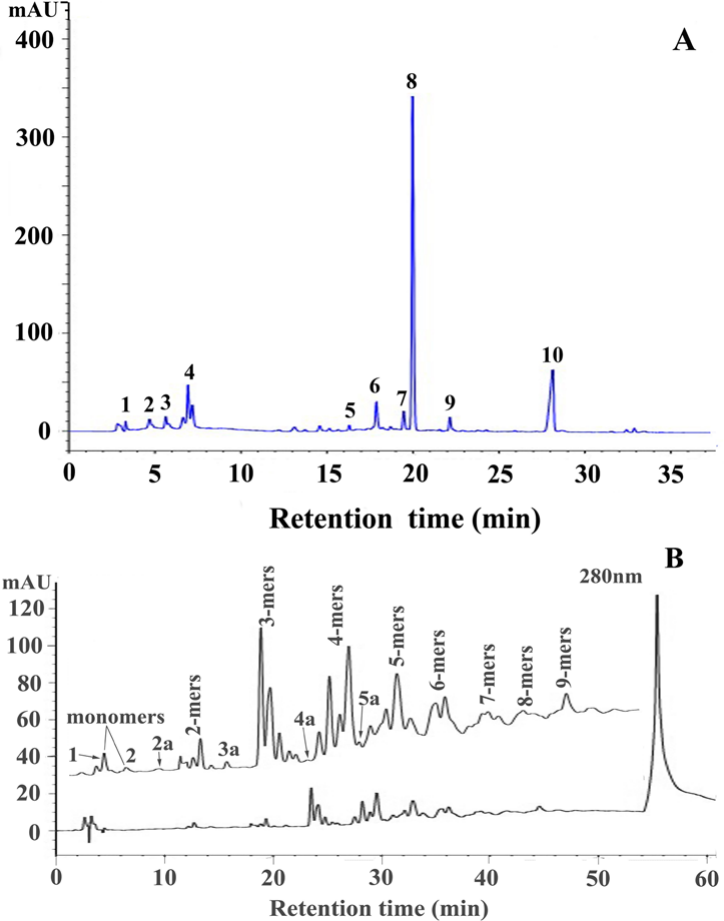
A, Reversed-phase HPLC-ESI-MS chromatograms of proanthocyanidins from *R*. *pulchrum* leaves after thiolytic degradation. Peaks 1, 2, 3, 4, 5, 6, 7, 8, 9, and 10 were gallocatechin, epigallocatechin, catechin, epicatechin, epigallocatechin/gallocatechin benzylthioether, A-type trimer benzylthioether, catechin benzylthioether, epicatechin benzylthioether, A-type dimer benzylthioether and epiafzelechin/afzelechin benzylthioether, and benzyl mercaptan, respectively. B, Normal-phase HPLC-ESI-MS chromatograms of *R*. *pulchrum* leaf proanthocyanidins.

Reversed-phase HPLC-ESI-MS analysis after thiolysis reaction was performed to obtain information on the construction unit of *R*. *pulchrum* proanthocyanidins. **Fig 3A** shows that the dominant peak detected was epicatechin benzylthioether (peak 8). In addtion, gallocatechin (peak 1), epigallocatechin (peak 2), catechin (peak 3), epicatechin (peak 4), epigallocatechin/gallocatechin benzylthioether (peak 5), A-type trimer benzylthioether (peak 6), catechin benzylthioether (peak 7), A-type dimer benzylthioether and epiafzelechin/afzelechin benzylthioether (peak 9), and benzyl mercaptan (peak 10) were also detected. These results indicated that *R*. *pulchrum* proanthocyanidins were mainly constituted of procyanidin which were composed of epicatechin unit. In addition, A-type linkage and B-type linkage were both involved in the formation of proanthocyanidins polymers.

### 3.3 Normal-phase HPLC-ESI-MS Analysis

The heterogeneity of proanthocyanidins can be further explained by normal-phase HPLC analysis. Although the polymers higher than 9-mers could not be separated and showed a single broad peak at the end of the chromatograms, the result obtained from normal-phase HPLC analysis (**Fig 3B, S1 Table**) revealed the presence of *R*. *pulchrum* proanthocyanidins with polymerization degree from monomers to nonamers. By comparison with standards and ESI-MS, peaks 1 and 2 were identified as epicatechin (289 Da, [M−H]^−^) and catechin (289 Da, [M−H]^−^), respectively. Peaks 2a (575 Da [M−H]^−^), 3a (863 Da, [M−H]^−^), 4a (1151 Da, [M−H]^−^), and 5a (1439 Da, [M−H]^−^) proved to be 2-mers, 3-mers, 4-mers, and 5-mers with A-type linkage, respectively **(S1 Table)**. Additionally, the higher oligomers were prone to form multiple charges **(Table in S1 Table)**. These results suggested the heterogeneity of *R*. *pulchrum* proanthocyanidins in their monomer units, distribution of polymerization degree, and interflavan linkage and offered a good complement to the MALDI-TOF MS analysis and the reversed phase HPLC-ESI-MS analysis.

### 3.4 Effect of *R*. *pulchrum* Leaf Proanthocyanidins on the Monophenolase Activity of Tyrosinase

**Fig 4:**
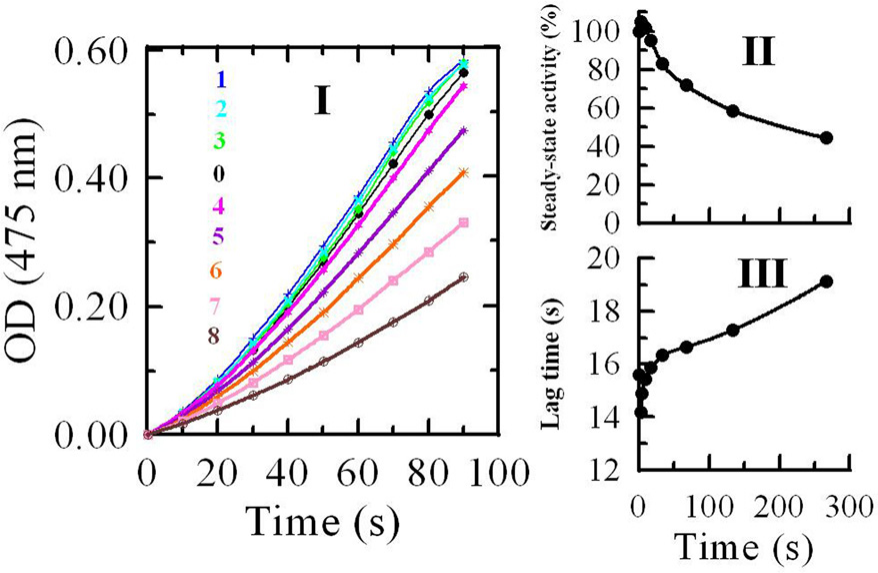
Inhibition effects of proanthocyanidins from *R*. *pulchrum* leaves on monophenolase activity of mushroom tyosinase: (I) Progress curves for the oxidation of L- tyrosine by the enzyme; (II) Effects on the steady-state rate and (III) on the lag time for the oxidation of tyrosine. The concentrations of proanthocyanidins for curves 0−8 were 0, 2, 4, 8, 16, 32, 64, 128, and 256 μg/mL, respectively.

The oxidation reaction of L-tyrosine catalyzed by mushroom tyrosinase was measured in the presence of different concentrations of *R*. *pulchrum* leaf proanthocyanidins (**Fig 4I)**. When the monophenolase activity of tyrosinase was tested, a lag period (lag time) **(Fig 4III)** was observed. The system reached steady-state rate **(Fig 4II)** after the lag time, which was obtained by extrapolation of the linear portion of the product accumulation curve to the abscissa. The slope and the intercept of the line denoted the steady-state rate and the lag time, respectively. The result obtained from **Fig 4II** revealed that the steady-state rate slightly increased in the presence of low concentration (≤10 ± 1 μg/mL) of *R*. *pulchrum* proanthocyanidins, this can be explained by the high absorption of products produced from the reaction of catechin/epicatechin or oligomeric proanthocganidins (exist in proanthocyanidins of *R*. *pulchrum* leaves) with tyrosinase at 475 nm [18]. The steady-state rate then decreased distinctly and was dose-dependent with the increase of proanthocyanidin concentration. The *IC*_*50*_ was estimated to be 200 ± 10 μg/mL. **Fig 4III** revealed that the proanthocyanidin slightly reduce the lag time of the enzyme in the existence of low concentration (≤10 ± 1 μg/mL) of *R*. *pulchrum* proanthocyanidins. This can be explained by the fact that tyrosinase needs 3,4-dihydroxyphenylalanine to start its monophenol oxidase activity, because of the necessity to reduce Cu^2+^ to Cu^+^ because only then the binuclear copper center type 3 of tyrosinase can bind dioxygen to oxidize tyrosine [24]. In the reactivity, the monomer (catechin/epicatechin) or oligomer (e.g. dimer, trimer) existed in *R*. *pulchrum* proanthocyanidins can take the place of 3,4-dihydroxyphenylalanine. They play the same role in the reaction system. In fact, catechin/epicatechin is also the substrate of tyrosinase [18]. The lag time extend with the increase of proanthocyanidin concentrations when the concentration ≥10 ± 1 μg/mL. It can be speculated that the influence of *R*. *pulchrum* proanthocyanidins on the lag time of the tyrosinase partly depends on the probability of the monomer (catechin/epicatechin) or oligomer binding to the enzyme. The probability decreased with the increase of *R*. *pulchrum* proanthocyanidin concentration. Therefore, it could be concluded *R*. *pulchrum* proanthocyanidins were good inhibitor of monophenolase activity.

### 3.5 Effects of *R*. *pulchrum* Leaf Proanthocyanidins on the Diphenolase Activity of Tyrosinase

**Fig 5:**
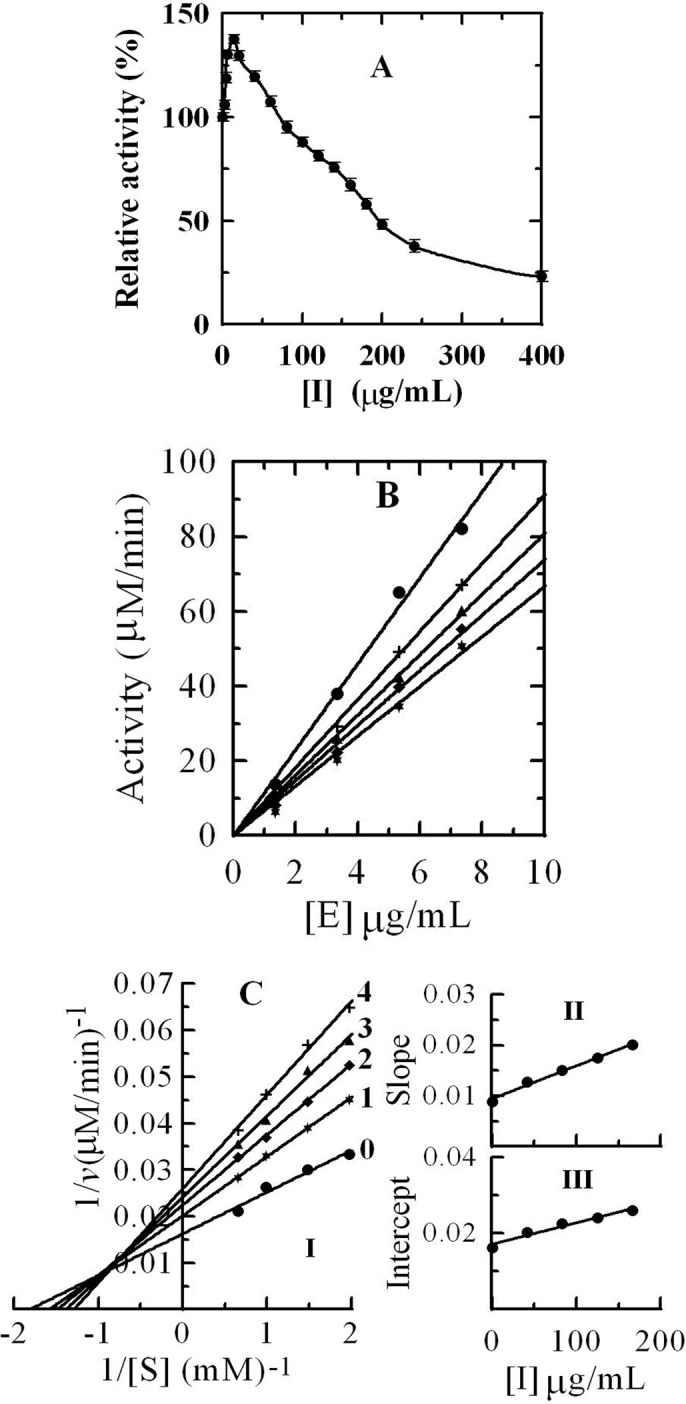
Inhibitory effects (A), inhibitory mechanism (B), and inhibitory type (C) of *R*. *pulchrum* leaf proanthocyanidins on the diphenolase activity of tyrosinase.

The effect of the *R*. *pulchrum* leaf proanthocyanidins on diphenolase activity of mushroom tyrosinase was shown in **Fig 5A**. The relative activity of the enzyme increased before reaching a maximum (137.7 ± 5.7% at 14 μg/mL) and decreased after increasing concentrations of the leaf proanthocyanidins. When the proanthocyanidin concentration reached 400 μg/mL, the relative activity decreased to 23.1 ± 2.1%. The inhibitor concentration leading to the loss of 50% enzyme activity was estimated to be 200 ± 10 μg/mL. The result was very similar to the finding in our previous report [18]. The reason of the increase of relative activity of the enzyme between 0 and 70 μg/mL may be the high absorption of products produced from the reaction of catechin/epicatechin or oligomeric proanthocganidins (exist in proanthocyanidins of *R*. *pulchrum* leaves) with tyrosinase at 475 nm. In fact, *R*. *pulchrum* leaf proanthocyanidins were potent inhibitor of diphenolase activity of the enzyme. They prevented the reaction of 3,4-dihydroxyphenylalanine and tyrosinase.

**Fig 5B** revealed the inhibition mechanism of the *R*. *pulchrum* proanthocyanidins on diphenolase activity. The results showed that the enzyme activity relied heavily on proanthocyanidins`concentration. The plots of the residual enzyme activity versus the concentrations of enzyme at different concentrations of proanthocyanidins gave a family of straight lines which passed through the origin, indicating that the inhibition was reversible.

The inhibition type analysis revealed that the oxidation of 3,4-dihydroxyphenylalanine catalyzed by diphenolase followed Michaelis-Menten kinetics. Plot of 1/*v* versus 1/[S] gave a family of lines with different slope and intercept, which intersected in the second quadrant, which indicated that *R*. *pulchrum* proanthocyanidins were mixed competitive inhibitors **(Fig 5C)**. The enzyme inhibitor constant (*K*_*I*_) was obtained from the plots of the slope versus the concentration of these compounds, and the enzyme–substrate complex (*K*_*IS*_), was obtained from the vertical intercept versus the concentration of these compounds. The values of *K*_*I*_ and *K*_*IS*_ were determined to be 145.3 and 304.7 μg/mL, respectively.

There has been an increasing interest in the use of plant extracts with tyrosinase inhibition activity. Comparing with lyophilized and methanolic extracts of *Aloe vera L*. *gel* [25] and red koji extracts [26], *R*. *pulchrum* leaf proanthocyanidins were much better tyrosine inhibitors. Though tyrosinase inhibitory properties were demonstrated with a number of benzaldehyde derivatives, none of them were considered to be safe in terms of practical use due to their lower activity and serious side effects [27]. Because of the fact that proanthocyanidins were already in use for different medicinal properties, it was apparent that these compounds could well be applied in pharmaceutical material and cosmetic additives.

### 3.6 Fluorescence Quenching Analysis

**Fig 6:**
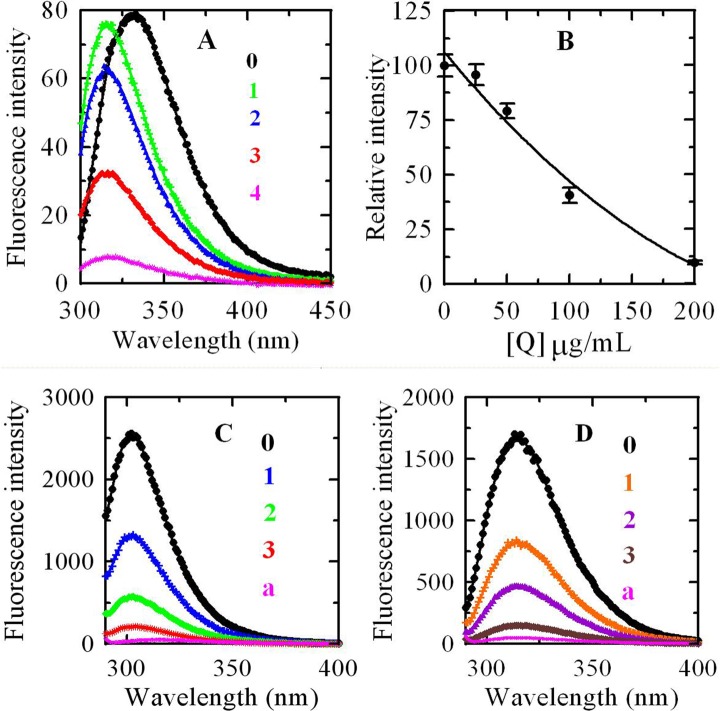
(A) Changes in intrinsic tyrosinase fluorescence at different concentrations of *R*. *pulchrum* proanthocyanidins. Emission spectra 0~4 of tyrosinase in the presence of proanthocyanidins are 0, 25, 50, 100 and 200 μg/mL, respectively; (B) Maximum florescence intensity changes; (C) Relative fluorescence intensities of L-tyrosine in solution with different concentrations of proanthocyanidins, the concentrations of proanthocyanidins of curves 0~3 were 0, 25, 50, and 100 μg/mL, respectively. Curve a was fluorescence intensity of proanthocyanidins when their concentration was 100 μg/mL.; (D) Relative fluorescence intensities of 3,4-dihydroxyphenylalanine in solution with different concentrations of proanthocyanidins, the concentrations of proanthocyanidins of curves 0~3 were 0, 25, 50, and 100 μg/mL, respectively. Curve a was fluorescence intensity of proanthocyanidins when their concentration was 100 μg/mL.

The interactions between tyrosinase (**Fig 6A and 6B**), L-tyrosine (**Fig 6C**), or 3,4-dihydroxyphenylalanine (**Fig 6D**) with *R*. *pulchrum* leaf proanthocyanidins were evaluated by fluorescence quenching analysis. The results showed that the addition of proanthocyanidins caused a dramatic decrease in the fluorescence emission intensity of these three compounds (**Fig 6**). A dose-response relationship was found when different concentrations of proanthocyanidins were added. These results provided important evidence that proanthocyanidins were effective binding agent of tyrosinase, L-tyrosine, and 3,4-dihydroxyphenylalanine. In addition, an obvious blue shift was also present in the fluorescence spectra of proanthocyanidins and tyrosinase (**Fig 6A**). The result indicated that proanthocyanidins induced the change of enzyme conformation after binding the enzyme molecule. It was concluded that the interactions of proanthocyanidins with enzyme and their substrate (L-tyrosine and 3,4-dihydroxyphenylalanine) were significant reasons to their strong inhibitory activity on the tyrosinase. Specifically, L-tyrosine and L-dihydroxyphenylalanine were proved to be hormone-like regulators of melanocyte functions [28], this revealed importance of *R*. *pulchrum* leaf proanthocyanidins in the regulation of melanocyte functions.

### 3.7 Interaction of Cu^2+^ and Proanthocyanidins

**Fig 7:**
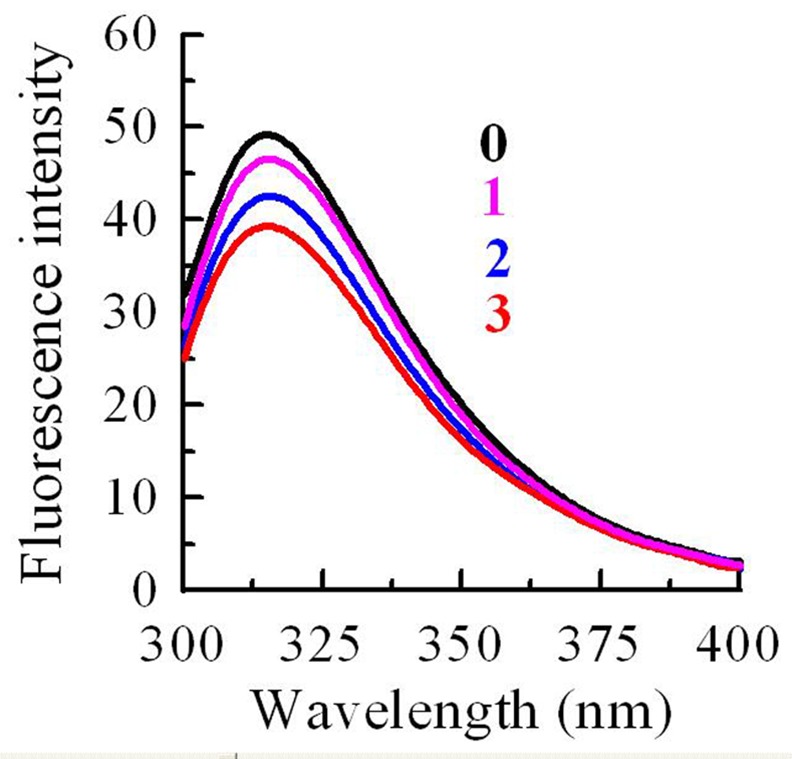
Interaction of Cu^2+^ and proanthocyanidins. The Cu^2+^ concentrations for curves 0~3 were 0, 12.5, 25, and 50 μM, respectively.

Interactions between *R*. *pulchrum* leaf proanthocyanidins and Cu^2+^ were studied by using fluorescence analysis. The intrinsic fluorescence intensity of proanthocyanidins increased with the addition of Cu^2+^ **(Fig 7)**_._ The Cu^2+^ concentrations for curve 0~3 were 0, 12.5, 25, and 50 μM, respectively. The results revealed the binding of Cu^2+^ and proanthocyanidins.

### 3.8 Molecular Docking Analysis

**Fig 8:**
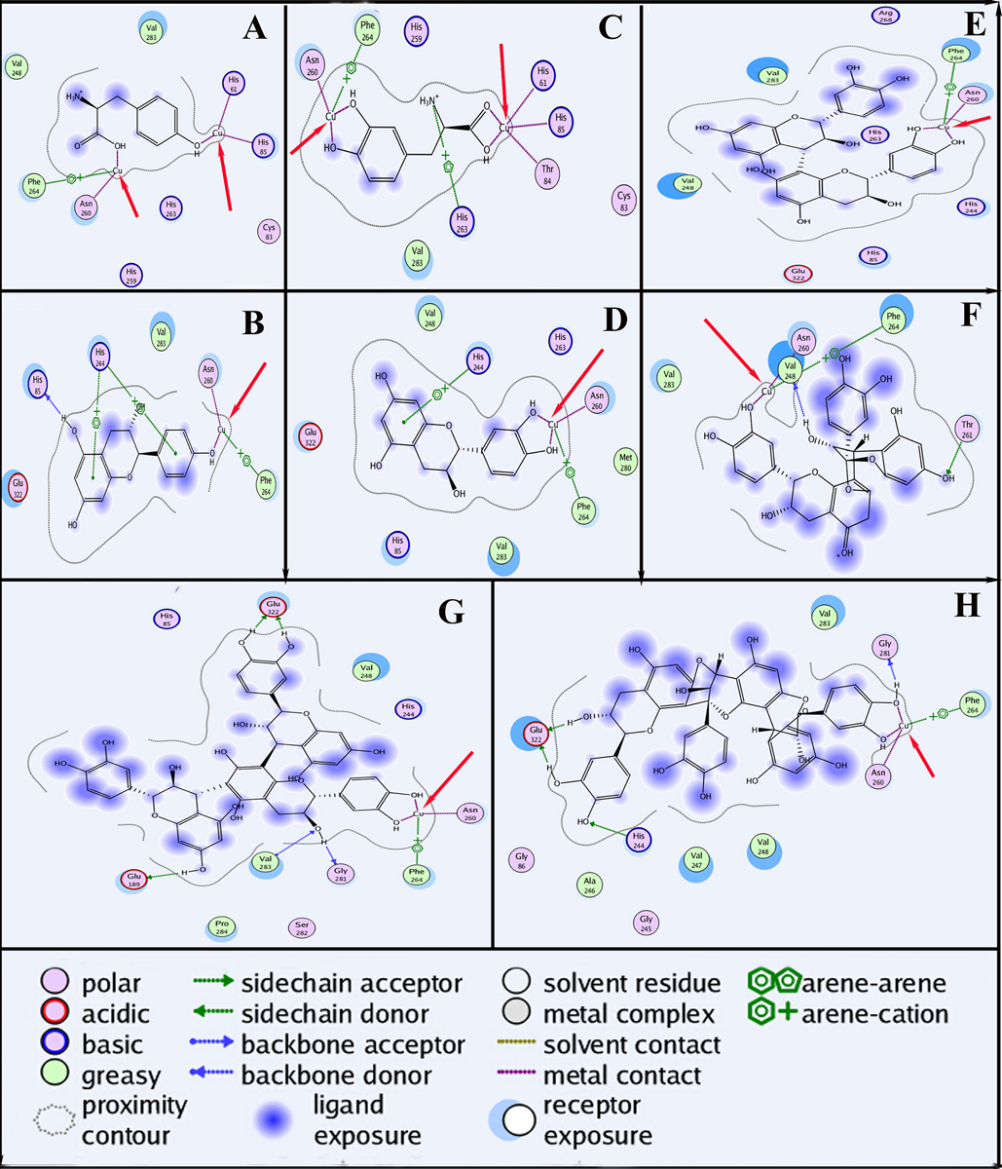
Binding mode of the lowest energy docked conformation found for the ligand with tyrosinase residues. The receptor exposure differences were shown by the size and intensity of the turquoise discs surrounding the residues. The red arrows indicated the interaction of the ligand with the copper iron. A, B, C, D, E, F, G, and H represented L-tyrosine, afzelechin/epiafzelechin, 3,4-dihydroxyphenylalanine, epicatechin/catechin, procyanidin dimer, A-type procyanidin dimer, procyanidin trimer, and A-type procyanidin trimer, respectively.

Docking simulations were carried out to work out the inhibition mechanism of proanthocyanidins on the tyrosinase. The docking modes of L-tyrosine, 3,4-dihydroxyphenylalanine, monomers of the proanthocyanidins (epi/catechin and epi/afzelelcin), proanthocyanidin dimmers, and proanthocyanidin trimers were examined in the enzyme catalytic site. The docked conformations revealed that all the compounds could interact with the dicopper irons of the enzyme (**Fig 8**). 3,4-dihydroxyphenylalanine could chelate two copper irons simultaneously (**Fig 8C**). Interestingly, the adjacent hydroxyl group on the B ring of epi/catechin (**Fig 8D**), B-type proanthocyanidin dimmer (**Fig 8E**), and proanthocyanidin trimers (**Fig 8G and 8H**) could chelate the copper ions in the enzyme active site, whereas L-Tyr (**Fig 8A**), epi/afzelelcin (**Fig 8B**) and A-type proanthocyanidin dimmer (**Fig 8F**) could not. The results revealed that the copper ion chelating ability of proanthocyanidins oligomer (e.g. monomers, dimers, or trimers) with adjacent hydroxyl group on the B ring may be the part mechanism to explain their inhibitory potency on the tyrosinase. However, we speculate proanthocyanidin polymer would be most probably dependent on the blockage of the reaction centre without carrying out the electron transfer rather than on chelating the tightly bound copper ions. It could also be caused by a conformational change of the apoenzyme.

## Conclusions

In conclusion, this study demonstrated that *R*. *pulchrum* leaf proanthocyanidins were complex mixtures of homo- and heteropolymers of procyanidin, prodelphinidin, propelargonidin, and their derivatives. They possessed structural heterogeneity in monomer units, interflavan linkage, substituent, and degree of polymerization. The enzyme assay experiments described here revealed that these compounds were potent, reversible and mixed competitive tyrosinase inhibitors. The interaction of proanthocyanidins with substrate (L-tyrosine and 3,4-dihydroxyphenylalanine) and copper ions located in the active site of tyrosinase were the important mechanisms to explain their efficient inhibition. The elucidation of the anti-tyrosinase mechanisms and activity was important because it provided a better understanding of *R*. *pulchrum* proanthocyanidins and was beneficial to the future design of novel and potent tyrosinase inhibitors.

## Supporting Information

S1 Table (DOC)
