# Supplementary material for: Proanthocyanidins Extracted from Rhododendron pulchrum Leaves as Source of Tyrosinase Inhibitors: Structure, Activity, and Mechanism
Source: PLoS One. 2015 Dec 29;10(12):e0145483. doi: 10.1371/journal.pone.0145483 (PMC4700988; doi:10.1371/journal.pone.0145483)
Supplement: S1 Table — (DOC) [file pone.0145483.s001.doc]

S1 Table. Masses and structural compositions of proanthocynidins from *R. pulchrum* leaves obtained by normal phase HPLC–ESI-MS and MALDI-TOF-MS analyses

| Polymers | Normal phase HPLC–ESI-MS | | MALDI-TOF-MS | | | | | |
| --- | --- | --- | --- | --- | --- | --- | --- | --- |
|  | Molecules ion | Structural compositions | Molecules ion | | Structural compositions | | | |
|  |  |  | Calculated masses | Observed masses | C/EC | GC/EGC | A/EA | -G  --  0  1  0  0  0  0  0  0  0  0  1  1  1  0  0  0  0  0  0  0  0  1  1  0  0  0  0  0  0  0  0  0  0  1  0  0  0  0  0  0  0  1  0  0  0  0  0  0  1  0  0  0  0  1  0  0  0  1  0  0  0  1  0  0  0  0  0  0  0  0  0  0  0 |
| Monomers | 289 | C/EC | -- | -- | -- | -- | -- |  |
| 2-mers | 575 | 2 (C/EC) + 1 (A-type) | 711 | 711 | 2 | 0 | 0 |  |
|  | 577 | 2 (C/EC) | 863 | 863 | 2 | 0 | 0 |  |
| 3-mers | 863 | 3 (C/EC) + 1 (A-type) | 979 | 979 | 2 | 0 | 1 |  |
|  |  |  | 981 | 981 | 2 | 0 | 1 |  |
|  |  |  | 983 | 983 | 2 | 0 | 1 |  |
|  |  |  | 997 | 997 | 3 | 0 | 0 |  |
|  |  |  | 999 | 999 | 3 | 0 | 0 |  |
|  | 865 | 3 (C/EC) | 1011 | 1011 | 3 | 1 | 0 |  |
|  |  |  | 1013 | 1013 | 3 | 1 | 0 |  |
|  |  |  | 1015 | 1015 | 3 | 1 | 0 |  |
|  |  |  | 1147 | 1147 | 3 | 0 | 0 |  |
|  |  |  | 1149 | 1149 | 3 | 0 | 0 |  |
|  |  |  | 1151 | 1151 | 3 | 0 | 0 |  |
| 4-mers | 1151 | 4 (C/EC) + 1 (A-type) | 1267 | 1267 | 2 | 0 | 2 |  |
|  |  |  | 1269 | 1269 | 3 | 0 | 1 |  |
|  |  |  | 1271 | 1271 | 3 | 0 | 1 |  |
|  |  |  | 1285 | 1285 | 4 | 0 | 0 |  |
|  |  |  | 1287 | 1287 | 4 | 0 | 0 |  |
|  | 1153 | 4 (C/EC) | 1301 | 1301 | 3 | 1 | 0 |  |
|  |  |  | 1303 | 1303 | 3 | 1 | 0 |  |
|  |  |  | 1423 | 1423 | 3 | 0 | 1 |  |
|  |  |  | 1437 | 1437 | 4 | 0 | 0 |  |
|  |  |  | 1439 | 1439 | 4 | 0 | 0 |  |
| 5-mers | 1439 | 5 (C/EC) + 1 (A-type) | 1537 | 1537 | 3 | 0 | 2 |  |
|  |  |  | 1539 | 1539 | 3 | 0 | 2 |  |
|  |  |  | 1555 | 1555 | 4 | 0 | 1 |  |
|  |  |  | 1557 | 1557 | 4 | 0 | 1 |  |
|  |  |  | 1559 | 1559 | 4 | 0 | 1 |  |
|  | 1441 | 5 (C/EC) | 1573 | 1573 | 5 | 0 | 0 |  |
|  |  |  | 1575 | 1575 | 5 | 0 | 0 |  |
|  |  |  | 1587 | 1587 | 4 | 1 | 0 |  |
|  |  |  | 1589 | 1589 | 4 | 1 | 0 |  |
|  |  |  | 1591 | 1591 | 4 | 1 | 0 |  |
|  |  |  | 1727 | 1727 | 5 | 0 | 0 |  |
| 6-mers | 1729 | 6 (C/EC) | 1845 | 1845 | 5 | 0 | 1 |  |
|  |  |  | 1847 | 1847 | 5 | 0 | 1 |  |
|  |  |  | 1859 | 1859 | 6 | 0 | 0 |  |
|  |  |  | 1861 | 1861 | 6 | 0 | 0 |  |
|  |  |  | 1863 | 1864 | 6 | 0 | 0 |  |
|  |  |  | 1877 | 1877 | 5 | 1 | 0 |  |
|  |  |  | 1879 | 1879 | 5 | 1 | 0 |  |
|  |  |  | 2015 | 2015 | 6 | 0 | 0 |  |
| 7-mers | 1008 | 7 (C/EC) | 2133 | 2133 | 6 | 0 | 1 |  |
|  |  |  | 2135 | 2135 | 6 | 0 | 1 |  |
|  |  |  | 2149 | 2149 | 7 | 0 | 0 |  |
|  |  |  | 2151 | 2152 | 7 | 0 | 0 |  |
|  |  |  | 2165 | 2165 | 6 | 1 | 0 |  |
|  |  |  | 2167 | 2167 | 6 | 1 | 0 |  |
|  |  |  | 2303 | 2303 | 7 | 0 | 0 |  |
| 8-mers | 1152 | 8 (C/EC) | 2421 | 2421 | 7 | 0 | 1 |  |
|  |  |  | 2423 | 2423 | 7 | 0 | 1 |  |
|  |  |  | 2439 | 2439 | 8 | 0 | 0 |  |
|  |  |  | 2455 | 2455 | 7 | 1 | 0 |  |
|  |  |  | 2591 | 2591 | 8 | 0 | 0 |  |
| 9-mers | 864 | 9 (C/EC) | 2711 | 2711 | 8 | 0 | 1 |  |
|  |  |  | 2727 | 2727 | 9 | 0 | 0 |  |
|  |  |  | 2743 | 2743 | 8 | 1 | 0 |  |
|  |  |  | 2879 | 2879 | 9 | 0 | 0 |  |
| 10-mers |  |  | 2999 | 3000 | 9 | 0 | 1 |  |
|  |  |  | 3015 | 3016 | 10 | 0 | 0 |  |
|  |  |  | 3031 | 3031 | 10 | 1 | 0 |  |
|  |  |  | 3167 | 3168 | 10 | 0 | 0 |  |
| 11-mers |  |  | 3303 | 3303 | 11 | 0 | 0 |  |
|  |  |  | 3319 | 3319 | 11 | 1 | 0 |  |
| 12-mers |  |  | 3591 | 3591 | 12 | 0 | 0 |  |
|  |  |  | 3607 | 3607 | 11 | 1 | 0 |  |
| 13-mers |  |  | 3879 | 3879 | 13 | 0 | 0 |  |
| 14-mers |  |  | 4167 | 4167 | 14 | 0 | 0 |  |
| 15-mers |  |  | 4455 | 4454 | 15 | 0 | 0 |  |
| 16-mers |  |  | 4743 | 4742 | 16 | 0 | 0 |  |
| 17-mers |  |  | 5031 | 5029 | 17 | 0 | 0 |  |
| 18-mers |  |  | 5319 | 5318 | 18 | 0 | 0 |  |
| 19-mers |  |  | 5607 | 5608 | 19 | 0 | 0 |  |
